# Professionalism and Dedication in Patients Reintegration

**DOI:** 10.25122/jml-2018-1001

**Published:** 2018

**Authors:** Victor Lorin Purcarea

The NATIONAL INSTITUTE FOR RECOVERY, PHYSICAL MEDICINE AND BALNEOCLIMATOLOGY is a prestigious institution under the aegis of the Ministry of Health, with a history and reputation recognized as valuable, useful, and professional. A reference methodological forum, the Institute develops three main activity directions: profile medical activity in Recovery and Balneology, research not only in the balneary-medical field but also in the natural therapeutic factors (water, air, soil, salines, pits, area of balneary protection, etc.) and activity of university education and medical postgraduate education in Physiotherapy and Balneary-Medical fields.

**Figure 1:**
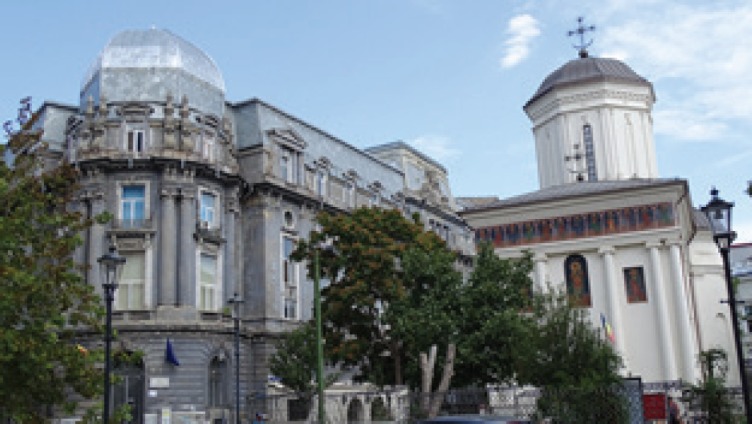
National Institute for Recovery, Physical Medicine, and Balneoclimatology

The institute was founded in 1924 under the name “Institute of Balneology”, by sustained strides of some outstanding personalities of that time – in the medical field or not - from the Ministry of Health, Red Cross in Romania, Romanian Patriarchy, Ministry of National Defense, etc.

The main objective of the Institute is the permanent improvement of the health state of the population through the recovery and balnear medical assistance, specifically targeting the functional aspect of integrating the patient in the daily and professional activity.

**Figure 2:**
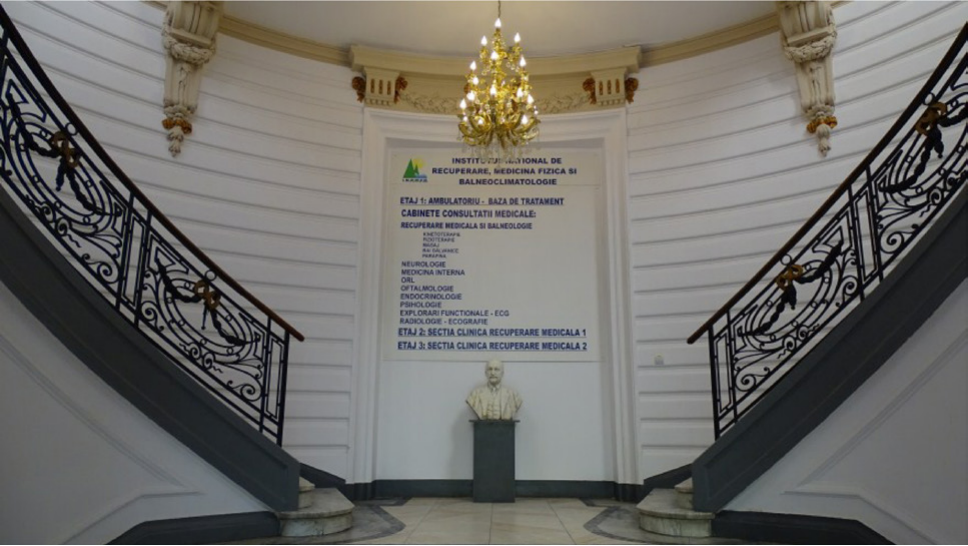
Main entrance of the Institute

It is an only profile hospital with 355 beds, Recovery, Physical Medicine and Balneoclimatology (including 45 beds in “Slӑnic Moldova” Balneary Sanatorium Section, but also 45 beds for day hospitalization). Moreover, the Institute has a complex activity in the balneary field through the accreditation and reaccreditation of the natural therapeutic factors at the level of the entire country. The National Institute for Recovery, Physical Medicine and Balneoclimatology is also one of the most important centers of professional training because it prepares the residents in the fields of Physical Medicine and Recovery, but also in other medical fields, and makes sure that the university courses of the students are in accordance with the university curriculum, through the collaboration with “Carol Davila” University of Medicine and Pharmacy, Bucharest.

**Figure 3,4:**
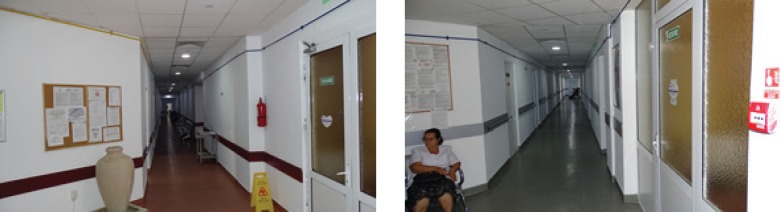
Hallway in the Institute

**Table 1:** Number of discharged patients, medium period of hospitalization and rate of using the hospital beds in each section

Indicator	No. of discharged patients	Medium period of hospitalization	Rate of using the hospital beds
Section 1	2151	11,16	87,42
Section 2	2106	11,40	87,43
Section 3	1384	11,54	87,31
Section 4	1536	13,38	86,40
SSSM	873	12,88	73,17
Total	8050	11,90	85,24

Due to the everyday life, stress, sedentariness and the diseases of the century we are living in, the Institute has become a recovery, physical medicine, and balneoclimatology (physical medicine and recovery) “assistance guide”, which is realized in the clinical sections and the specialty ambulatory services.

Regarding healthcare, the Institute ensures the complex treatment of patients with multiple dysfunctional problems resulted from locomotor, neurological, posttraumatic, rheumatologic, peripheral-vascular, dysmetabolic and other conditions. The patients benefit from balneo-physiotherapy and kinesiotherapy treatments aiming at the substantial improvement of the functional deficits and the clinical picture characteristic for different pathologies.

The number of patients discharged from hospital varies from year to year because great efforts have been made to cover all the cases under contract with the National Health Insurance House by using the waiting list for the patients who can be programmed, sometimes this number of discharges exceeding the number of cases under contract with the National Health Insurance House.

**Figure 5:**
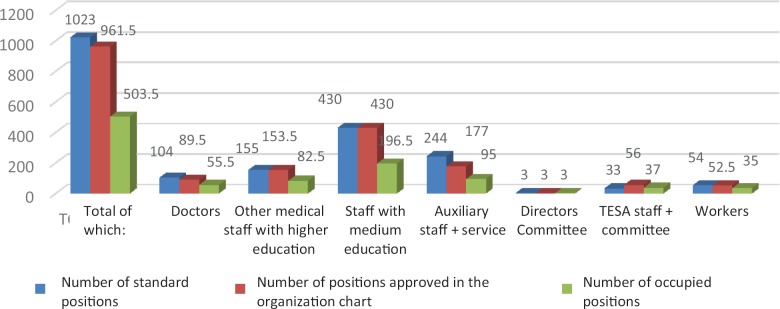
Structure of the staff

**Figure 6:**
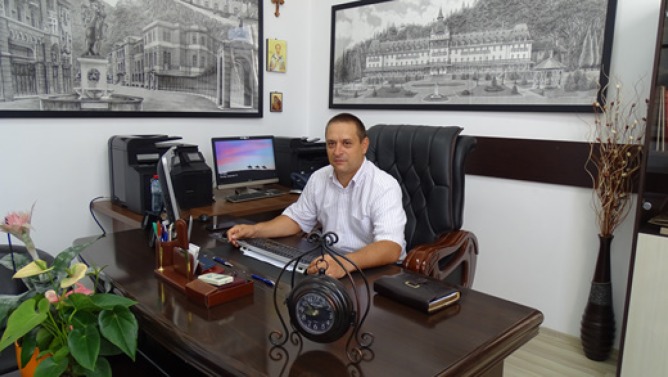
Horia Lӑzӑrescu, MD, PhD, Manager of the Institute

Regarding the medical devices, the hospital has performing equipment specific for medical recovery, a lot of which has been purchased in the last 3 years.

The National Institute for Recovery, Physical Medicine and Balneoclimatology is equipped with two Siemens Fusion radiology and medical imagining devices, one being installed and used in Sfântul Dumitru Center, by the staff of the two clinics (Medical Recovery-Orthopedics and Traumatology and Recovery and Physical Medicine and Balneology II), and the other, in the Center located on 11A Ion Mihalache Blvd., by the staff in the Clinic of Recovery, Physical Medicine and Balneology III and the Clinic of Medical Recovery – Neurology.

3 performing ultrasound devices with options for abdominal, cardiac and soft tissues exploration are used in the two above-mentioned centers.

A DEXA osteodensiometry device is used in Sfântul Dumitru Center, where approximately 500 patients are examined annually.

A device for medical robotics, used especially for the reeducation of walking in patients with post neurologic affections (strokes, vertebra-medullary traumas, and so forth) is used in the Center located on 11A Ion Mihalache Blvd.

The laboratories of medical analyses are equipped with devices needed to ensure the optimal criteria of functioning and to close contracts with the National Health Insurance House.

The kinesiotherapy rooms benefit from new generation equipment for all the types of specific medical procedures.

The treatment bases in the clinical but also in the ambulatory sections have performing devices that ensure the rise in the quality of medical services of the Institute.

What should be mentioned is that there is a hydro kinesiotherapeutic recovery basin for complex medical procedures with a natural factor in the area (spring no. 3) in the treatment base of the external section in Slӑnic Moldova.

The Institute has the most important clinical base and academic staff involved in the profile medical education programs at all levels (students, residents, specialists, physio- kinesiotherapists, kinesiotherapists, and medium education specialty staff).

The concerted efforts of the members from leadership and the employees, who work in this extremely necessary Institute, contributing with professionalism and dedication to the growth of the quality of life of the patients and their integration in their families and implicitly in society, represent the premises for creating an “Excellence Center” in the National Institute for Recovery, Physical Medicine and Balneoclimatology.

